# Cognitive Reactivity, Implicit Associations, and the Incidence of Depression: A Two-Year Prospective Study

**DOI:** 10.1371/journal.pone.0070245

**Published:** 2013-07-26

**Authors:** Anne-Wil Kruijt, Niki Antypa, Linda Booij, Peter J. de Jong, Klaske Glashouwer, Brenda W. J. H. Penninx, Willem Van der Does

**Affiliations:** 1 Institute of Psychology, Leiden University, Leiden, The Netherlands; 2 Department of Psychiatry, University of Montreal, Montreal, Canada; 3 Sainte-Justine Hospital Research Center, Montreal, Canada; 4 Department of Psychiatry, McGill University, Montreal, Canada; 5 Department of Clinical Psychology, University of Groningen, Groningen, The Netherlands; 6 Department of Psychiatry, University Medical Center Groningen, Groningen, The Netherlands; 7 Department of Psychiatry, Vrije Universiteit Medical Center, Amsterdam, The Netherlands; 8 Department of Psychiatry, Leiden University Medical Center, Leiden, The Netherlands; 9 Leiden Institute for Brain and Cognition, Leiden, The Netherlands; The University of Queensland, Australia

## Abstract

**Background:**

Cognitive reactivity to sad mood is a vulnerability marker of depression. Implicit self-depressed associations are related to depression status and reduced remission probability. It is unknown whether these cognitive vulnerabilities precede the first onset of depression.

**Aim:**

To test the predictive value of cognitive reactivity and implicit self-depressed associations for the incidence of depressive disorders.

**Methods:**

Prospective cohort study of 834 never-depressed individuals, followed over a two-year period. The predictive value of cognitive reactivity and implicit self-depressed associations for the onset of depressive disorders was assessed using binomial logistic regression. The multivariate model corrected for baseline levels of subclinical depressive symptoms, neuroticism, for the presence of a history of anxiety disorders, for family history of depressive or anxiety disorders, and for the incidence of negative life events.

**Results:**

As single predictors, both cognitive reactivity and implicit self-depressed associations were significantly associated with depression incidence. In the multivariate model, cognitive reactivity was significantly associated with depression incidence, together with baseline depressive symptoms and the number of negative life events, whereas implicit self-depressed associations were not.

**Conclusion:**

Cognitive reactivity to sad mood is associated with the incidence of depressive disorders, also when various other depression-related variables are controlled for. Implicit self-depressed associations predicted depression incidence in a bivariate test, but not when controlling for other predictors.

## Introduction

The central thesis of cognitive theory of depression is that dysfunctional cognitions render an individual vulnerable to developing depressive episodes [1]. Dysfunctional cognitions are thought to arise from negative belief systems that develop during childhood. These systems can remain relatively inactive until later in life, for instance when an individual encounters a situation (e.g., a demanding boss) that resembles the circumstances that led to the belief system (e.g., demanding parents) [1]. Psychotherapy directed at modifying dysfunctional belief systems, cognitive behavioral therapy (CBT), is more effective at preventing relapse than pharmacotherapy [2], [3], [4], [5], providing indirect evidence for the causal relation between dysfunctional cognitions and depression risk. However, dysfunctional cognitions, prominent during depressed states, tend to normalize during remission [6], [7], [8], [9], [10], and research yielded mixed results regarding the question whether negative cognitions are antecedents, consequences or by-products of depression [10], [11], [12], [13].

Findings became more consistent when it was realized that negative cognitions might go undetected unless primed or activated by stress or a dysphoric mood state [14], [15], [16]. Cognitive reactivity to sad mood is the extent to which dysfunctional cognitions become activated when an individual experiences mild sadness. Several lines of evidence support the position that cognitive reactivity is a vulnerability marker of depression. Cognitive reactivity is higher in remitted depressed than never-depressed individuals [17], [18], [19], [20], [21], and it is associated with biological indices of depression vulnerability such as response to tryptophan depletion [22] and the polymorphism in the promotor region of the serotonin transporter gene SLC6A4 [23]. Moreover, cognitive reactivity may have prognostic value: high cognitive reactivity following treatment predicts shorter time to relapse or recurrence [21], [24]. It is unknown however, whether cognitive reactivity is also a risk factor for depression incidence, i.e. whether higher cognitive reactivity precedes first onset of depression.

Another strategy to make dysfunctional cognitions measurable is to rely on laboratory tests instead of self-report. One of these is the Implicit Associations Test (IAT) [25], [26], a reaction time test developed in social psychology. In this test, the relative speed with which an individual is able to generate the same motor responses to stimuli representing two different concepts, is used as an index of the strength of the individual’s association between these concepts [25]. Implicit associations between the concepts ‘self’ and ‘depressed’ are stronger in currently depressed patients and remain elevated when depression is in remission [27]. Implicit self-depressed associations mediate the relationship between childhood emotional abuse and depression symptom severity [28], and are associated with suicidal ideation [29]. In currently depressed individuals, the strength of implicit self-depressed associations was inversely associated with the chance of achieving remission within a two-year period [30]. It has not yet been tested whether the strength of implicit self-depressed associations predicts depression incidence.

In the current study, we tested the hypotheses that cognitive reactivity and the strength of implicit self-depressed associations precede and predict the first onset of depressive disorders. A sample of never-depressed individuals was followed over a period of two years. Using multivariate binary logistic regression analysis, the prognostic values of cognitive reactivity and implicit self-depressed associations were assessed and tested against the contributions of a number of background variables and established risk factors of depression.

## Methods

### Participants

All data were collected within the Netherlands Study of Depression and Anxiety (NESDA). This is a large longitudinal cohort study investigating a range of factors implicated in the onset and course of depression and anxiety disorders [31]. The cohort of 2,981 participants consists of individuals with a current or lifetime diagnosis of depression or anxiety, and a number of never-depressed and/or never-anxious participants who were included as healthy controls at baseline. Participants were between 18 and 65 years old, and recruited through mental health organizations, primary care practices and in the general population. Detailed information on in-exclusion criteria, participant flow, and sample characteristics is provided by Penninx et al. [32]. For the current study, all individuals who had never experienced major depression or dysthymia at baseline were selected.

### Measures

#### Depression incidence

The main outcome measure, was determined using the Composite International Diagnostic Interview (CIDI; World Health Organization (WHO) Version 2.1) at the two years follow-up assessment. Incidence of a major depressive episode or a diagnosis of dysthymia was coded for as 1, versus 0 for no incidence. The CIDI is a standardized interview that assesses the, current and past, presence of psychiatric diagnoses as described in the DSM-IV [33]. Trained interviewers administered the CIDI [32].

#### Cognitive reactivity to sad mood

Was assessed with the Leiden Index of Depression Sensitivity – revised (LEIDS-r). The LEIDS-r has 34 items that assess the extent to which dysfunctional cognitions are activated when an individual experiences mild dysphoria [34], [35]. Two example items are: ‘When in a sad mood, I more often think about how my life could have been different’ (rumination subscale) or ‘When I feel sad I feel more like breaking things’ (aggression subscale). Items are scored on a 5-point Likert scale ranging from ‘not at all’ (0) to ‘very strongly’ (4). The LEIDS-r has a total score, and six subscales assessing cognitive reactivity related to Aggression, Hopelessness/Suicidality, Acceptance/Coping, Control/Perfectionism, Risk Aversion, and Rumination on Sadness. LEIDS-r scores were found to be associated with depression history over and above rumination [36], to be associated with genetic markers of depression [23], [37], [38], and with response to tryptophan depletion, reflecting biological vulnerability to depression [22]. Moreover, treatment and other longitudinal studies support the validity of the LEIDS-r as a measure of depression vulnerability [39], [40], [41], [42].

#### Implicit self-depressed associations (ISDA)

Were measured using the Implicit Association Test (IAT) [25], [43]. In this test participants have to respond to words presented on a display by pressing one of two response buttons. Each word belongs to either one of two concept-pairs. In this particular IAT, one set of stimulus words represented either elated (e.g., valuable, optimistic) or depressed (e.g., useless, pessimistic) concepts, whereas another set represented either the self (e.g., me, myself) or others (e.g., you, they)^1^. Within each test block, two concepts share the same button. The combination of concepts sharing a button was varied over blocks, i.e. within one block ‘elation’ and ‘self’ shared a button while in another block ‘depression’ and ‘self’ shared a button. The difference in reaction times between these two blocks indicates the strength of the implicit association between the concepts ‘self’ and ‘depression’. Raw IAT response times were transformed into the D600-measure recommended by Greenwald et al. [44] and others [45]. The D600-algorithm prescribes that: (i) data from two practice blocks (20 trials each) and two test blocks (60 trials each) are used; (ii) trials with reaction times above 10,000 ms are discarded; (iii) error trials are replaced with the mean reaction times of the correct responses in the block in which the error occurred, plus a penalty of 600 ms; (iv) response times for the self - elated blocks are subtracted from the response times for the self - depressed blocks (separately for practice and test blocks); (v) these difference scores are divided by their pooled standard deviation, and then averaged [44]. Lower values represent stronger implicit self-depressed associations.

#### Demographic information

Including gender, age, and years of education was obtained in an interview.

#### The presence of a lifetime anxiety diagnosis

Was determined with the lifetime version 2.1 of the Composite International Diagnostic Interview (CIDI; World Health Organization (WHO)).

#### Family history of anxiety and/or depression

Was assessed using the self report family tree method [46]. A positive family history was defined as reporting having at least one sibling or parent diagnosed with a depressive disorder, an anxiety disorder, or both.

#### Negative life events

That occurred during baseline and the two years follow-up session were indexed using the Brugha questionnaire [47]. This questionnaire assessed the occurrence of twelve negative life events such as illness or injuries to the self or close friends and relatives, loss of friends, relatives or partners, loss of job or housing, and being victimized by theft or assault.

#### Depressive symptoms

Were assessed with the 30-item Inventory of Depression Symptomatology – Self Report (IDS-SR) [48]. Each item is presented as four statements regarding the severity of a symptom, which are associated with scores ranging from 0 to 3.

#### Neuroticism

Was assessed with the NEO-FFI [49]. The neuroticism scale consists of twelve items that index the tendency to experience negative emotional states. Items were scores on a 5-point Likert scale ranging from ‘strongly disagree’ (0) to ‘strongly agree’ (4).

### Procedure

Baseline measures were assessed within a single 3 to 5 hours session. The follow-up measures (CIDI and Brugha) were again assessed within a single session, two years following baseline [32].

### Ethics Statement

The protocol for the NESDA study was approved by the Ethical Review Board of the Vrije Universiteit Medical Centre in Amsterdam (VUMC), as well as by the review boards of the participating medical centers (Leiden University Medical Center (LUMC) and University Medical Center Groningen (UMCG)). All participants received full verbal and written information about the study, and written informed consent was obtained at the start of baseline assessment. Participants received a 15-euro gift certificate and compensation of travel costs [32].

### Statistical Analyses

#### Binary logistic regression

Was used to assess predictive values for the incidence of depressive disorders over the course of the two-years. Following bivariate analyses for each of the predictor variables, multivariate binary logistic regression was used to assess the combined prognostic value of the variables. Age, sex, years of education, history of anxiety disorders, family history of anxiety and/or depression, number of negative life events between baseline and outcome measurement (NLE), baseline depressive symptom level (IDS-SR), and neuroticism (NEO-FFI subscale) were entered in a first block. Implicit self-depressed associations and cognitive reactivity were added in respectively blocks 2a and 2b. The third and final block contained all variables. Regression outcomes are presented as odds ratios and their associated 95% confidence intervals. Odds ratios represent the change in probability of the outcome event to occur, associated with a single unit increase on the predictor’s scale.

#### Predictor probability plots

Were drawn to provide an additional impression of the possible clinical usefulness for all continuous predictors that were found to be significantly associated with depression incidence in the bivariate analyses. These were based on the regression formula:
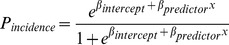



Using values for *β* derived from bivariate binary logistic regression analyses, the values x on the instrument’s scale associated with.00,.25,.50,.75, and 1.00 predicted probability of depression incidence are represented on the x-axis.

## Results

### Participant Flow

The NESDA cohort (n = 2,981) contained 1,008 individuals who had never experienced a depressive disorder at baseline. Of these, 174 persons had missing data on one or more measures and were excluded from the sample: LEIDS-r scores were missing for 85 participants, IAT for 24, and baseline severity or personality measures for 12 participants. Ninety participants dropped out following baseline, thus for these no information on the outcome measure of depression incidence was available.

Consequently, 834 participants were included in the present analyses: 596 were recruited from primary care, 76 from specialized mental health care, and 162 from the general population.

### Analyses of Drop-outs

In- and excluded participants were compared on all variables used in the analysis, plus recruitment origin (general population, primary, or mental health care). The excluded group differed significantly from the included group on variables years of education (*t*_(1006)_ = −2.405, *p* = .016), IDS-SR (*t*_(1000)_ = 3.132, *p* = .002), neuroticism (*t*_(1002)_ = 2.296, *p* = .022), and the presence of a lifetime anxiety diagnosis (χ^2^_(1)_^ = ^11.619, *p* = .001). A previous paper, reporting analyses of attrition over this period in detail, indicated that within the entire NESDA sample lower education and higher baseline symptoms were associated with attrition [50]. Importantly, the in- and excluded participants did not differ significantly with respect to the main variables of interest, cognitive reactivity (*t*_(921) = _−1.42, *p* = .155), and implicit self depressed associations (*t*_(981)_ = −.189, *p* = .850). A trend towards a difference was found on depression incidence (χ^2^_(1)_^ = ^3.48, *p* = .061), in line with an association between higher baseline symptom levels and attrition. See supplementary table S1 for all comparisons between in- and excluded participants.

### Main Analyses

Demographic and clinical characteristics at baseline for groups with and without depressive disorder at follow-up are presented in table 1.

**Table 1:** Sample characteristics.

	DD incidence(*n* = 84)		no DD incidence(*n* = 750)		total(*n* = 834)		
	*n*	*%*	*n*	*%*	*n*	*%*	
recruitment site							
primary care	547	72.9	49	58.3	596	71.5	
mental health care	58	7.7	18	21.4	76	9.1	
general population	145	19.3	17	20.2	162	19.4	
female	58	69.0	474	63.2	532	63.8	
lifetime anxiety	52	61.9	223	29.7	275	33.0	
family history	67	79.8	530	70.7	597	71.6	
	***M***	***sd***	***M***	***sd***	***M***	***sd***	***range***
age	40.1	14.9	41.6	14.4	41.5	14.4	18–65
education (yrs)	11.9	3.4	12.8	3.2	12.7	3.3	5–18
*n* NLE	2.1	1.7	1.4	1.2	1.4	1.3	0–9
IDS-SR	21.2	10.6	10.4	8.7	11.5	9.4	0–48
neuroticism	36.4	7.7	28.9	8.1	29.6	8.4	12–56
ISDA	0.25	0.39	0.39	0.38	0.37	0.38	−0.92–1.27
CR	35.0	16.5	20.0	14.3	21.5	15.2	0–98

DD incidence = incidence of depressive disorders between baseline and two-years follow-up, family history = family history of anxiety and/or depressive disorders, NLE = negative life events, IDS-SR = Inventory of Depressive Symptomatology – Self Report; Neuroticism = neuroticism subscale of the NEO-FFI; ISDA = implicit self-depressed associations (IAT); CR = Cognitive Reactivity (LEIDS-R).

The correlations between depression incidence and all predictor variables were calculated (see table S2). The largest correlation (*r_s_* = .73) was found between neuroticism (NEO-FFI) and baseline depressive symptom levels (IDS-SR). Most other correlations were significant but small to moderate in size (*r_s_* = .04 – *r_s_* = .57). Therefore multicollinearity was unlikely, which was confirmed by inspection of the variance inflation factor values, which ranged from 1.03 to 2.66.

Bivariate binary regression analyses showed that, as single predictors, most variables, including cognitive reactivity and implicit self-depressed associations, were significantly associated with first-onset of depressive disorder, see table 2.

**Table 2:** Bivariate binary logistic regression for depression incidence.

	OR	95% CI	
gender	1.30	[0.80–2.11]	
age	0.99	[0.98–1.01]	
education (yrs)	0.92*	[0.86–0.99]	
anxiety diagnosis	3.84***	[2.41–6.13]	
family history anx/dep	1.64	[0.94–2.85]	
*n* NLE	1.46***	[1.25–1.71]	
IDS-SR	1.11***	[1.08–1.13]	
neuroticism	1.11***	[1.08–1.15]	
ISDA	0.41**	[0.23–0.73]	
CR	1.06***	[1.05–1.08]	

OR = Odds Ratio; 95% CI = 95% confidence interval.

* *= p*<.05;

** = *p*<.01;

*** = *p*<.001.

NLE = Negative Life Events; IDS-SR = Inventory of Depressive Symptomatology – Self Report; Neuroticism = neuroticism subscale of the NEO-FFI; CR = Cognitive Reactivity (LEIDS-R); ISDA = Implicit Self-Depressed Associations (IAT).

Predictor probability plots are presented in the supplementary materials (figure S1) for the bivariately associated continuous measures. From these probability plots it can be assessed that baseline symptom levels (IDS-SR), cognitive reactivity (LEIDS-R), and to a lesser extent the number of negative life events, perform relatively well in predicting depression incidence.

The third and final block of the multivariate binary logistic regression analysis is presented in table 3 (for the entire multivariate analysis see table S3). Within this model, baseline depressive symptom levels (IDS), cognitive reactivity (CR), and the number of negative life events during the study period (NLE) were significant predictors of depressive disorder incidence over the course of two years. Implicit self-depressed associations were not found to be predictive of first onset of depressive disorders when other predictors were controlled for.

**Table 3:** Multivariate binary logistic regression for depression incidence – final block.

model χ^2^∶ 117.90***	OR	95% CI
gender	0.99	[0.57–1.73]
age	0.98	[0.97–1.00]
education (yrs)	0.95	[0.88–1.03]
lifetime anxiety	1.55	[0.88–2.72]
family history anx/dep	0.87	[0.46–1.63]
* n* NLE	1.34***	[1.16–1.65]
IDS-SR	1.08***	[1.04–1.12]
neuroticism	0.99	[0.94–1.04]
ISDA	1.00	[0.50–2.01]
CR	1.03***	[1.01–1.05]

OR = Odds Ratio; 95% CI = 95% confidence interval.

* *= p*≤.05;

** = *p*≤.01;

*** = *p*≤.001.

NLE = Negative Life Events; IDS-SR = Inventory of Depressive Symptomatology – Self Report; Neuroticism = neuroticism subscale of the NEO-FFI; ISDA = Implicit Self-Depressed Associations (IAT); CR = Cognitive Reactivity (LEIDS-R).

### Additional Analyses

Previous papers assessing the predictive validity of self-depressed associations also assessed explicit self-depressed associations [30]. Adding a block 2c, containing the baseline predictors plus explicit self-depressed associations, did not yield a significant outcome for explicit self-depressed associations (OR = 0.90 [0.66–1.22] *n.s.,* block 2c χ^2^ = .45, *n.s.*), nor did adding this predictor to block 3. Other studies hypothesized and found effects pertaining to specific subscales of the LEIDS-R [40]. We assessed our model with LEIDS-R total score replaced by each of the six subscales. The control/perfectionism, risk avoidance, and the rumination subscales were significant predictors within the model. The models containing the risk avoidance or rumination subscale may explain slightly more variance than the model containing the LEIDS-R total scale (model χ^2^ were 120.99 (risk avoidance), and 120.42 (rumination), versus 117.90 (LEIDS-R total)). These differences are small and it is not possible to formally test whether the fit of two non-nested models differ significantly.

## Discussion

The current study assessed the two-year prognostic value for depression incidence of two, prospectively assessed, cognitive risk factors in a large population-based sample. As single predictors, cognitive reactivity and implicit self-depressed associations were significantly associated with depression incidence. When other predictors were taken into account, cognitive reactivity remained associated with depression incidence. Contrary to our hypothesis, implicit self-depressed associations did not. In the multivariate model, baseline depressive symptoms and the number of negative life events between baseline and follow-up were also significantly associated. These measures predicted depression onset over predictors such as neuroticism and the lifetime presence of an anxiety disorder.

The LEIDS-R does not assess the current activation of negative cognitions, but rather an individual’s assessment of the extent to which these become more activated during sad mood. This is a crucial difference if one wants to test the assumption that latent negative cognitions predict depression incidence [14]. The current findings support cognitive models stating that certain depression-related cognitions precede first onset of depression. Contrary to our hypothesis, implicit self-depressed associations did not contribute to the prediction in the multivariate analysis. Previous NESDA studies reported stronger self-depressed associations in remitted depressed individuals [27], and a positive relationship between the number of prior episodes and the strength of individuals’ self-depressed associations [51]. Combined with the current result, this suggests that implicit self-depressed associations may not precede first-onset depression, but rather represent a cognitive scar that emerges in response to a depressive episode, rendering remitted patients more vulnerable for new depressive episodes.

Both baseline depressive symptoms and cognitive reactivity significantly add to the multivariate model, despite their moderate correlation of.52. This indicates that these two measures assess distinctive constructs, at least to a certain degree. Neuroticism, an established predictor of depression risk, did not significantly add to the prediction, probably due to shared variance with baseline symptom levels. The correlation between these two measures was.73. Shared variance between implicit self-depressed associations and baseline depressive symptoms may also account for the finding that implicit self-depressed associations do not add to the prediction of depressive incidence in the multivariate model, even though the (highly significant) correlation was only −.28.

To get an impression of the possible prognostic usability of the assessed instruments, graphical displays of the predictions derived from the bivariate regression analysis were provided in the supplementary material (figure S1). These were based on bivariate analyses, as we were interested to assess predictions derived from single instruments. Visual inspection makes it clear that cognitive reactivity (LEIDS-R) is relatively well suited to discern amongst levels of incidence probability.

A main limitation of these findings is limited generalizability. It should be noted that the NESDA sample is a ‘risk enriched’ sample, recruited in a large part among depressive and anxious patients [32]. This may explain the relatively high incidence of 10%, considering that the 12-month incidence of MDD in the Netherlands has been estimated at 2.7% [52]. This may also explain why a family history of anxiety and/or depression was reported by as many as 72% of our sample.

The current study tested the hypothesis that two cognitive measures predict depression incidence over a two-year period. From a theoretical perspective it would be interesting to assess the prognostic value of cognitive measures over a longer period. The currently presented two-year prediction may, however, be more interesting from a practical clinical perspective.

In conclusion, cognitive reactivity to sad mood was associated with the incidence of depressive disorders. This association remained when various other risk factors of depression are controlled for. Implicit self-depressed associations were also significant predictors of depression incidence, but only when bivariately tested.

## Supporting Information

Figure S1 **Predicted probability plots.** Derived from bivariate regression analyses. Grey areas represent 95% confidence intervals. For questionnaires, the x-axis extends the possible range. For the measures education and implicit self-depressed associations, the observed range is represented on the x-axis. Note that a history of anxiety diagnoses was also found to be significantly associated with depression incidence, yet, being a dichotomous variable, not represented here.
(DOC)

Table S1 **Comparison of in- and excluded participants on demographic and clinical variables.** * several participants had missing data on more than one measure, hence the numbers do not add up to the total of 174 participants excluded. DD = depressive disorder, MDD = major depressive disorder, family history = family history of anxiety and/or depressive disorders, NLE = negative life events, IDS-SR = Inventory of Depressive Symptomatology – Self Report; Neuroticism = neuroticism subscale of the NEO-FFI; ISDA = implicit self-depressed associations (IAT); CR = Cognitive Reactivity (LEIDS-R).
(DOCX)

Table S2 **Correlation matrix.** * = *p*≤.05; ** = *p*≤.01; *** = *p*≤.001. DD incidence = incidence of depressive disorders, family history = family history of anxiety and/or depression, NLE = Negative Life Events; IDS-SR = Inventory of Depressive Symptomatology – Self Report; Neuroticism = neuroticism subscale of the NEO-FFI; ISDA = Implicit Self-Depressed Associations (IAT); CR = Cognitive Reactivity (LEIDS-R).
(DOCX)

Table S3 **Multivariate binary logistic regression for depression incidence.** OR = Odds Ratio; 95% CI = 95% confidence interval. NLE = Negative Life Events; IDS-SR = Inventory of Depressive Symptomatology – Self Report; Neuroticism = neuroticism subscale of the NEO-FFI; ISDA = Implicit Self-Depressed Associations (IAT); CR = Cognitive Reactivity (LEIDS-R).
(DOCX)
