# Supplementary figures and images for: Cognitive Reactivity, Implicit Associations, and the Incidence of Depression: A Two-Year Prospective Study
Source: PLoS One. 2013 Jul 26;8(7):e70245. doi: 10.1371/journal.pone.0070245 (PMC3724814; doi:10.1371/journal.pone.0070245)

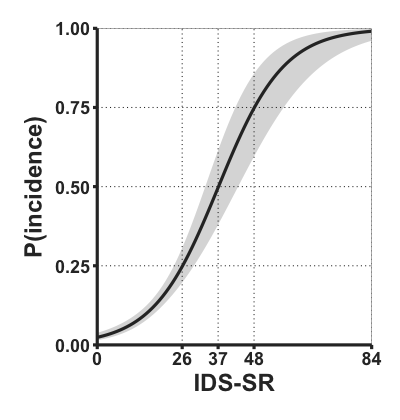

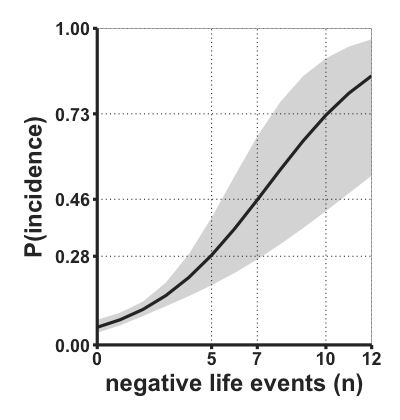

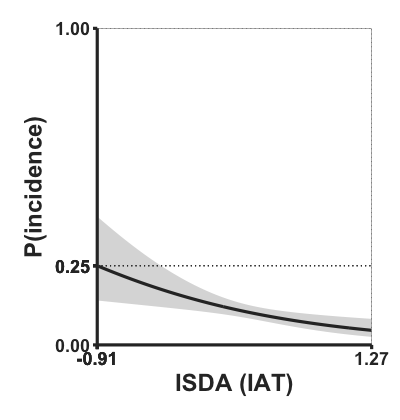

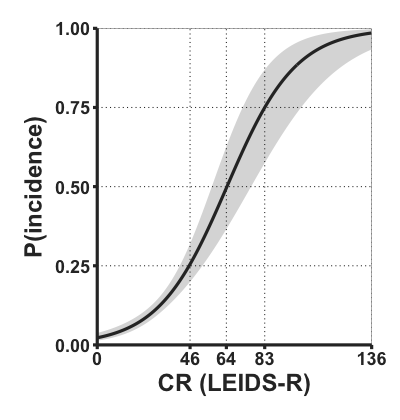

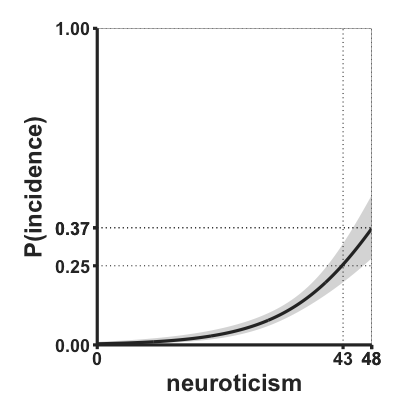

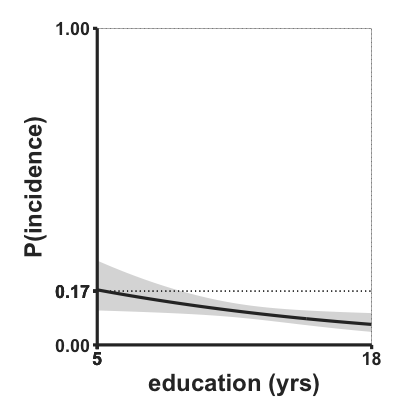

Supplement: Figure S1 — Predicted probability plots. Derived from bivariate regression analyses. Grey areas represent 95% confidence intervals. For questionnaires, the x-axis extends the possible range. For the measures education and implicit self-depressed associations, the observed range is represented on the x-axis. Note that a history of anxiety diagnoses was also found to be significantly associated with depression incidence, yet, being a dichotomous variable, not represented here. (DOC) [file pone.0070245.s001.doc]
