# Supplementary material for: Cognitive Reactivity, Implicit Associations, and the Incidence of Depression: A Two-Year Prospective Study
Source: PLoS One. 2013 Jul 26;8(7):e70245. doi: 10.1371/journal.pone.0070245 (PMC3724814; doi:10.1371/journal.pone.0070245)
Supplement: Table S1 — Comparison of in- and excluded participants on demographic and clinical variables. * several participants had missing data on more than one measure, hence the numbers do not add up to the total of 174 participants excluded. DD = depressive disorder, MDD = major depressive disorder, family history = family history of anxiety and/or depressive disorders, NLE = negative life events, IDS-SR = Inventory of Depressive Symptomatology – Self Report; Neuroticism = neuroticism subscale of the NEO-FFI; ISDA = implicit self-depressed associations (IAT); CR = Cognitive Reactivity (LEIDS-R). (DOCX) [file pone.0070245.s002.docx]

|  | included  (*n* = 834) | | excluded  (*n*= 174) | | |  | | |
| --- | --- | --- | --- | --- | --- | --- | --- | --- |
|  | *n* | *%* | *n* | *%* | missing*** | *χ^2^* | *p* |  |
| recruitment site |  |  |  |  |  | 0.93 | .630 |  |
| primary care | 596 | 71.5 | 118 | 76.8 |  |  |  |  |
| mental health care | 76 | 9.1 | 18 | 10.3 |  |  |  |  |
| general population | 162 | 19.4 | 38 | 21.8 |  |  |  |  |
| DD incidence |  |  |  |  | 90 | 0.79 | .672 |  |
| dysthymic disorder | 4 | 0.5 | 1 | 0.6 |  |  |  |  |
| MDD | 80 | 9.6 | 13 | 7.5 |  |  |  |  |
| female | 532 | 63.8 | 101 | 58.0 |  | 2.03 | .154 |  |
| lifetime anxiety | 275 | 33.0 | 81 | 46.6 |  | 11.62 | .001 |  |
| family history | 597 | 71.6 | 122 | 70.5 | 1 | 0.08 | .778 |  |
|  | *M* | *sd* | *M* | *sd* |  | *t* | *p* |  |
| age | 41.5 | 14.4 | 41.3 | 14.2 |  | -0.12 | .906 |  |
| education (yrs) | 12.7 | 3.3 | 12.1 | 3.1 |  | -2.49 | .014 |  |
| *n* NLE | 1.4 | 1.3 | 1.7 | 1.3 | 90 | 1.81 | .071 |  |
| IDS-SR | 11.5 | 9.4 | 14.0 | 10.4 | 6 | 3.13 | .002 |  |
| neuroticism | 29.6 | 8.3 | 31.3 | 9.1 | 5 | 2.30 | .022 |  |
| ISDA | .37 | .38 | .37 | .38 | 25 | -0.19 | .850 |  |
| CR | 21.5 | 15.2 | 19.1 | 16.3 | 85 | -1.42 | .155 |  |
