# Supplementary material for: Cognitive Reactivity, Implicit Associations, and the Incidence of Depression: A Two-Year Prospective Study
Source: PLoS One. 2013 Jul 26;8(7):e70245. doi: 10.1371/journal.pone.0070245 (PMC3724814; doi:10.1371/journal.pone.0070245)
Supplement: Table S2 — Correlation matrix. * = p≤.05; ** = p≤.01; *** = p≤.001. DD incidence = incidence of depressive disorders, family history = family history of anxiety and/or depression, NLE = Negative Life Events; IDS-SR = Inventory of Depressive Symptomatology – Self Report; Neuroticism = neuroticism subscale of the NEO-FFI; ISDA = Implicit Self-Depressed Associations (IAT); CR = Cognitive Reactivity (LEIDS-R). (DOCX) [file pone.0070245.s003.docx]

|  | 1. | 2. | 3. | 4. | 5. | 6. | 7. | 8. | 9. | 10. | 11. |
| --- | --- | --- | --- | --- | --- | --- | --- | --- | --- | --- | --- |
| 1. DD incidence | - |  |  |  |  |  |  |  |  |  |  |
| 2. sexe | .037 | - |  |  |  |  |  |  |  |  |  |
| 3. age | -.033 | -.076^*^ | - |  |  |  |  |  |  |  |  |
| 4. education (yrs) | -.083^*^ | -.052 | -.057 | - |  |  |  |  |  |  |  |
| 5. lifetime anxiety | .206^***^ | .072^*^ | .031 | -.074^*^ | - |  |  |  |  |  |  |
| 6. family history | .061 | .078^*^ | -.117^***^ | -.071^*^ | .137^***^ | - |  |  |  |  |  |
| 7*. n* NLE | .172^***^ | .060 | .027 | -.056 | .075^*^ | .104^**^ | - |  |  |  |  |
| 8. IDS-SR | .344^***^ | .123^***^ | .073^*^ | -.192^***^ | .482^***^ | .151^***^ | .119^**^ | - |  |  |  |
| 9. neuroticism | .271^***^ | .129^***^ | -.107^**^ | -.114^***^ | .471^***^ | .196^***^ | .099^**^ | .730^***^ | - |  |  |
| 10. ISDA | -.105^**^ | -.095^**^ | -.047 | .029 | -.222^***^ | -.054 | -.046 | -.277^***^ | -.307^***^ | - |  |
| 11. CR | .296^***^ | .061 | -.075^*^ | .057 | .334^***^ | .163^***^ | .132^***^ | .519^***^ | .573^***^ | -.231^***^ | - |
