# Supplementary material for: Cognitive Reactivity, Implicit Associations, and the Incidence of Depression: A Two-Year Prospective Study
Source: PLoS One. 2013 Jul 26;8(7):e70245. doi: 10.1371/journal.pone.0070245 (PMC3724814; doi:10.1371/journal.pone.0070245)
Supplement: Table S3 — Multivariate binary logistic regression for depression incidence. OR = Odds Ratio; 95% CI = 95% confidence interval. NLE = Negative Life Events; IDS-SR = Inventory of Depressive Symptomatology – Self Report; Neuroticism = neuroticism subscale of the NEO-FFI; ISDA = Implicit Self-Depressed Associations (IAT); CR = Cognitive Reactivity (LEIDS-R). (DOCX) [file pone.0070245.s004.docx]

|  |  | OR | 95% CI | χ^2^ | *p* |
| --- | --- | --- | --- | --- | --- |
| block 1 |  |  |  | 105.90 | <.001 |
|  | gender | 0.92 | [0.54 – 1.58] |  | .762 |
|  | age | 0.98 | [0.97 – 1.00] |  | .069 |
|  | education (yrs) | 0.98 | [0.90 – 1.06] |  | .539 |
|  | lifetime anxiety | 1.59 | [0.91 – 2.76] |  | .103 |
|  | family history anx/dep | 0.93 | [0.50 – 1.74] |  | .824 |
|  | *n* NLE | 1.41 | [1.18 – 1.68] |  | <.001 |
|  | IDS-SR | 1.09 | [1.05 – 1.13] |  | <.001 |
|  | neuroticism | 1.02 | [0.97 – 1.06] |  | .492 |
| block 2a |  |  | 1-2a: | .003 | .953 |
|  | gender | 0.92 | [0.53 – 1.59] |  | .758 |
|  | age | 0.98 | [0.97 – 1.00] |  | .069 |
|  | education (yrs) | 0.98 | [0.90 – 1.06] |  | .538 |
|  | lifetime anxiety | 1.58 | [0.91 – 2.76] |  | .105 |
|  | family history anx/dep | 0.93 | [0.50 – 1.74] |  | .825 |
|  | *n* NLE | 1.41 | [1.18 – 1.68] |  | <.001 |
|  | IDS-SR | 1.09 | [1.05 – 1.13] |  | <.001 |
|  | neuroticism | 1.02 | [0.97 – 1.06] |  | .503 |
|  | ISDA | 0.98 | [0.49 – 1.95] |  | .953 |
| block 2b |  |  | 1-2b: | 11.99 | .001 |
|  | gender | 0.99 | [0.57 – 1.72] |  | .968 |
|  | age | 0.98 | [0.97 – 1.00] |  | .081 |
|  | education (yrs) | 0.95 | [0.88 – 1.03] |  | .250 |
|  | lifetime anxiety | 1.55 | [0.88 – 2.71] |  | .130 |
|  | family history anx/dep | 0.87 | [0.46 – 1.63] |  | .659 |
|  | *n* NLE | 1.38 | [1.16 – 1.65] |  | <.001 |
|  | IDS-SR | 1.08 | [1.04 – 1.12] |  | <.001 |
|  | neuroticism | 0.99 | [0.94 – 1.04] |  | .615 |
|  | CR | 1.03 | [1.01 – 1.05] |  | .001 |
| block 3 |  |  | 2a-3: | 11.99 | .001 |
|  |  |  | 2b-3: | 0.00 | .997 |
|  | gender | 0.99 | [0.57 – 1.73] |  | .968 |
|  | age | 0.98 | [0.97 – 1.00] |  | .083 |
|  | education (yrs) | 0.95 | [0.88 – 1.03] |  | .250 |
|  | lifetime anxiety | 1.55 | [0.88 – 2.72] |  | .131 |
|  | family history anx/dep | 0.87 | [0.46 – 1.63] |  | .659 |
|  | *n* NLE | 1.34 | [1.16 – 1.65] |  | <.001 |
|  | IDS-SR | 1.08 | [1.04 – 1.12] |  | <.001 |
|  | neuroticism | 0.99 | [0.94 – 1.04] |  | .618 |
|  | ISDA | 1.00 | [0.50 – 2.01] |  | .997 |
|  | CR | 1.03 | [1.01 – 1.05] |  | .001 |
| model |  |  |  | 117.90 | <.001 |
